# Stress-State-Dependent Reinforcement of Cement-Stabilized Soil Using Waste Brick Powder and Glass Fiber

**DOI:** 10.3390/ma19163410

**Published:** 2026-08-11

**Authors:** Xiaosan Yin, Md Mashiur Rahman, Jian Wang, Alip Mohammed

**Affiliations:** 1 School of Civil Engineering, Zhengzhou University, Zhengzhou 450002, China; 2 School of Intelligent Construction and Civil Engineering, Zhongyuan University of Technology, Zhengzhou 451191, China; 2024209001@zut.edu.cn (M.M.R.); 2024109018@zut.edu.cn (J.W.); 3 School of Resources and Safety Engineering, University of Science and Technology Beijing, Beijing 100083, China; alipmohaammed5@gmail.com

**Keywords:** cement-stabilized soil, waste brick powder, glass fiber, mechanical properties, microstructure, stress-state dependence, sustainable composites

## Abstract

Cement-stabilized soil is widely used for ground improvement but suffers from brittleness and low tensile strength. This study investigates combined modification of cement-stabilized soil with waste brick powder (WBP, 5%) and glass fiber (GF, 0–2.0%) to enhance mechanical performance and microstructural integrity. Unconfined compressive strength (UCS), splitting tensile strength (STS), and qualitative scanning electron microscopy were evaluated at 3, 14, and 28 days. Results reveal stress-state-dependent reinforcement: UCS peaked at 2.0% GF with gains of 18.1%, 7.3%, and 9.1%, while STS maximized at 1.5% GF (43% increase), declining 14.3% at 2.0% due to fiber agglomeration. Post-peak ductility improved markedly, with the residual strength ratio increasing from 12.4% to 43.2% and the ductility index from 1.18 to 1.85. Microstructural analysis suggests that uniform fiber dispersion enables crack bridging and interfacial load transfer, whereas agglomeration creates localized weak zones. Preliminary assessment indicates that WBP substitution may avoid approximately 85 kg CO_2_eq per cubic meter by valorizing construction waste. These preliminary laboratory findings suggest potential stress-state-dependent dosage trends for sustainable cement-stabilized soil composites, pending further validation.

## 1. Introduction

The rapid advancement of urban–rural integration in China has intensified challenges of high energy consumption and environmental pollution in the construction industry, necessitating green, low-carbon building materials [1]. Cement-stabilized soil (cement-soil) is widely adopted for treating problematic soft soil subgrades characterized by high compressibility and low strength [2], yet it suffers from low tensile strength, high brittleness, and excessive stiffness, restricting engineering applicability under tensile or flexural loading [3].

**Figure 1:**
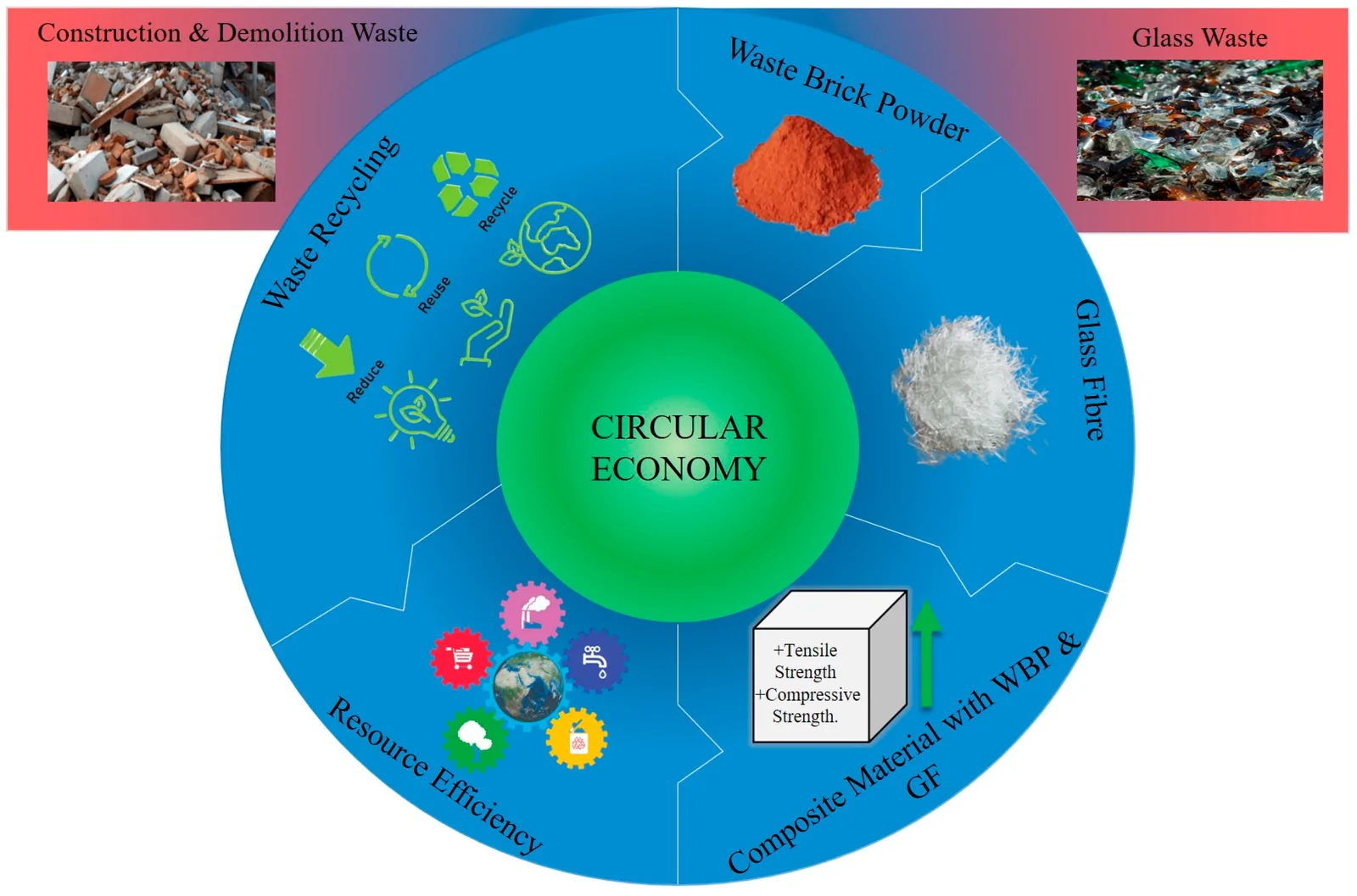
Sustainability rationale for glass fiber–reinforced waste brick powder cement-soil.

Fiber reinforcement addresses these deficiencies: incorporating discrete fibers enhances compressive and tensile strength, improves toughness, and mitigates brittle failure [4,5,6]. Glass fiber (GF) is particularly notable for its high tensile strength, elastic modulus, and chemical stability [7,8]. Concurrently, construction generates substantial brick debris; processed into waste brick powder (WBP), this material serves as a supplementary cementitious material with pozzolanic reactivity [9,10,11]. The combined use of WBP and GF presents a dual benefit—improved mechanical performance and advanced waste recycling—aligning with circular economy principles (Figure 1).

Research on fiber-reinforced cementitious materials evolved from alkali-free glass fiber introduction in the late 1950s [12] to industrial production in the 1970s [13] and fiber–matrix interaction studies in the early 1990s [14]. With growing emphasis on sustainable construction, research expanded to include WBP utilization. International studies confirmed GF effectiveness in enhancing tensile strength and toughness in cement-soil composites [7,8,15], with systematic investigations into fiber content, geometry, and curing effects [4,5,16]. Wang et al. (2022) demonstrated that combined cement–fiber action significantly improves strength and deformation behavior [16].

Chinese research began in the early 2000s [17,18], with systematic advances during the 2010s demonstrating GF enhancement of compressive strength through crack restriction and tensile strength through fiber-bridging mechanisms [2,19,20]. Triaxial tests revealed stabilizing effects under varying confining pressures [21], while investigations identified optimal fiber dosages for soil stabilization applications [22,23,24]. However, combined WBP–GF interaction within a unified composite remains unexamined [25], and gaps persist regarding reinforcement mechanisms and long-term durability [26,27]. Although recent studies have examined PVA fiber reinforcement in clayey soils [28], ecological sustainability across global construction industries [29], and composite-cemented backfill for ultra-deep foundations [30], none have integrated WBP and GF within a single cement-stabilized soil system. This study elucidates the mechanical and microstructural behavior of WBP–GF cement-stabilized soil. The objectives are: (1) quantify GF effects on strength development across curing stages; (2) identify fiber content optimizing peak strength and post-cracking toughness; and (3) analyze failure behavior and microstructural evolution, clarifying fundamental reinforcement mechanisms in this sustainable composite system.

## 2. Materials and Methods

### 2.1. Materials

#### 2.1.1. Soil

**Figure 2:**
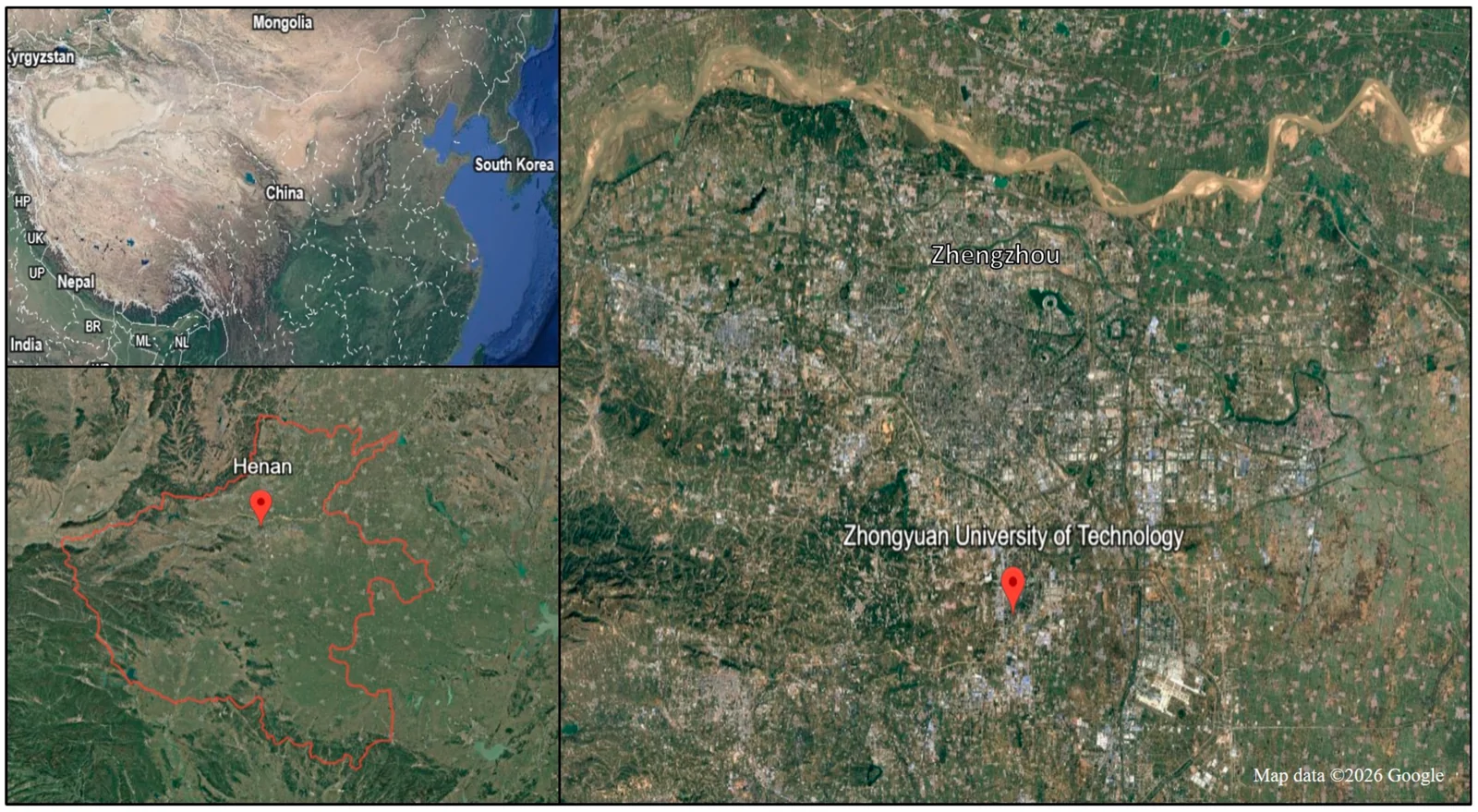
Geolocation of the soil sampling site.

Soft clay collected from a construction site at the North Campus of Zhongyuan University of Technology, Zhengzhou, Henan Province, China, was used as the base material for this study (shown in Figure 2). The soil contained a small proportion of brick fragments originating from on-site construction activities. Prior to testing, the material was air-dried, crushed using a laboratory ball mill, and sieved through a 1 mm mesh to obtain a uniform particle size distribution. Representative subsamples were then tested to determine basic index properties, including natural moisture content, liquid limit, plastic limit, and density, following the procedures specified in GB/T 50123-2019 (Chinese Standard for Geotechnical Testing Methods) [31]. The resulting physical and mechanical properties of the soil are summarized in Table 1.

#### 2.1.2. Cementitious Binder

Ordinary Portland cement of strength class PO 42.5 was used as the cementitious binder in this study. The cement exhibited a compressive strength of 23.4 MPa at 3 days and 42.8 MPa at 28 days. The physical and mechanical properties of the cement are summarized in Table 2.

To align with circular economy principles, waste brick powder (WBP) was produced from construction waste bricks collected from local demolition sites. The bricks were crushed using a laboratory crusher and sieved through a 1 mm mesh to obtain WBP with controlled particle size. The resulting powder was air-dried prior to specimen preparation to minimize the influence of residual moisture. WBP was incorporated as a supplementary cementitious material at a dosage of 5% by mass of the dry soil. Local tap water was used for all mixing and curing procedures.

#### 2.1.3. Glass Fiber Reinforcement

Alkali-free glass fiber was used as the reinforcing material and was cut into uniform lengths of 6 mm, with a nominal fiber diameter of 7 μm. The fibers were supplied as a commercial construction-grade alkali-free product. The geometric properties of the glass fiber are summarized in Table 3.

### 2.2. Test Specimen Design

#### 2.2.1. Specimen Preparation and Mix Design

Mix proportions were designed in accordance with the Chinese standard JGJ/T 233-2011 (Specification for mix proportion design of cement-soil) [32]. The target specimen geometry was a 70.7 mm cube, which is the standard specimen shape specified in JGJ/T 233-2011 and JTG E51-2009 (Test methods of materials stabilized with inorganic binders for highway engineering) [33] for unconfined compressive strength testing of cement-stabilized soils. While ASTM D2166 [34] specifies cylindrical specimens, the 70.7 mm cube was adopted to maintain consistency with Chinese engineering practice and to facilitate direct comparison with domestic literature.

The batch quantities presented in Table 4 represent the total weighed masses required to cast three 70.7 mm cubic specimens per mix, with a correction factor of 1.3 applied to the cementitious materials (Portland cement and WBP) to account for preparation losses during mixing, molding, and compaction. The water content was determined based on workability requirements for the fiber-reinforced soil mixture, accounting for the absorption characteristics of the air-dried soil (1.2% moisture content) and the glass fiber. Consequently, the total mixing water (652 g per batch) exceeds the nominal cement hydration water requirement. All materials were weighed using an electronic balance with 0.1 g precision. The dry soil, cement, and WBP were first blended in a planetary mixer for 2 min at low speed. Glass fiber was then gradually added and mixed for an additional 3 min to promote dispersion. Water was finally introduced, and the mixture was blended for 4 min. The fresh mixture was immediately placed into 70.7 mm steel cube molds in two layers, with each layer subjected to vibration until visible air bubbles ceased. Excess material was struck off, and the molds were sealed and placed in a standard curing room (20 ± 2 °C, relative humidity ≥ 95%) for 24 h before demolding.

During specimen preparation, soil, waste brick powder, cement, and glass fiber were added sequentially to a mechanical mixer and dry-mixed until a uniform distribution was achieved. Water was then gradually introduced while mixing continued to ensure homogeneous consistency. To minimize material segregation, mixing was briefly interrupted to manually homogenize material adhering to the base of the mixing container.

**Figure 3:**
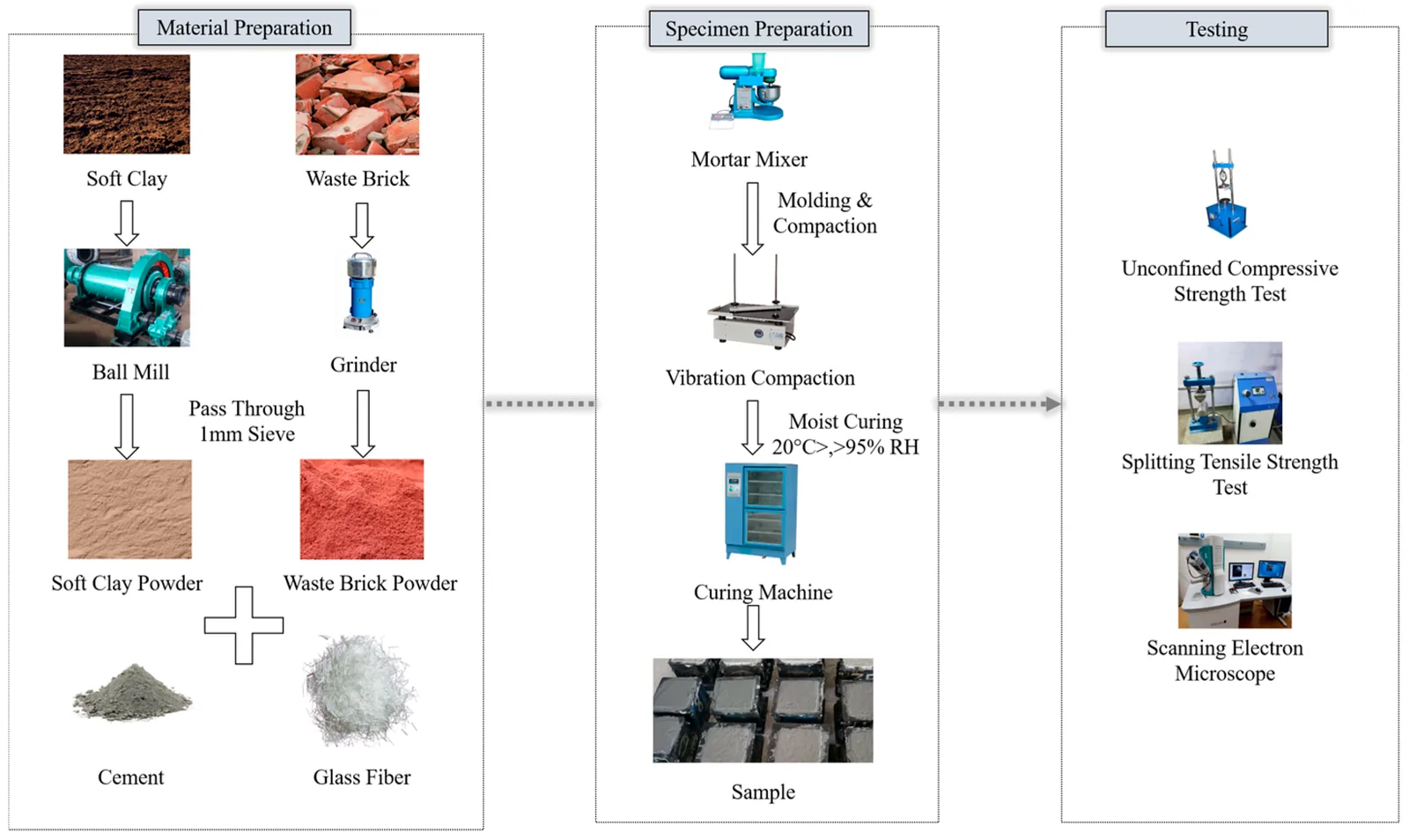
Specimen preparation and testing procedure.

The prepared mixture was placed into lubricated cubic molds in two layers, with each layer subjected to vibration until visible air bubbles ceased. After mould filling, specimen surfaces were leveled and sealed with plastic film to prevent moisture loss during initial setting. Specimens were carefully demolded after initial hardening to avoid the formation of microcracks that could influence subsequent mechanical testing. The demolded specimens were grouped and cured under standard conditions at a temperature of 20 °C and a relative humidity exceeding 95% for curing periods of 3, 14, and 28 days. Mechanical testing was conducted immediately upon completion of curing. For each mixture and curing age, three replicate specimens were fabricated and tested (*n* = 3), yielding a total of 45 specimens (5 mixtures × 3 ages × 3 replicates). An additional three specimens per mixture were reserved for microstructural analysis, bringing the total to 60 specimens. Figure 3 summarizes the four-stage specimen preparation workflow from material proportioning through to curing.

#### 2.2.2. Testing Apparatus

Sample preparation and testing were conducted using standard laboratory equipment suitable for soil stabilization studies. Soil and waste brick materials were crushed and sieved using mechanical grinding and screening equipment to obtain controlled particle sizes. Mixing was performed using a planetary mixer capable of sequential dry and wet mixing to ensure uniform fiber dispersion. Specimens were molded in cubic molds with a side length of 70.7 mm and compacted using a vibration table to eliminate entrapped air.

Curing was carried out in a controlled-environment chamber maintained at a temperature of 20 °C and a relative humidity exceeding 95%. Mechanical testing was conducted using a microcomputer-controlled testing machine capable of applying controlled loading rates and recording load–displacement responses. Auxiliary equipment, including precision balances and standard measuring tools, was used to ensure accurate material proportioning and specimen preparation.

#### 2.2.3. Testing Methodology

Unconfined compressive strength and splitting tensile strength tests were conducted on cubic specimens with dimensions of 70.7 mm × 70.7 mm × 70.7 mm. For unconfined compression testing, specimens were loaded at a constant displacement rate of 1 mm/min in accordance with ASTM D2166/D2166M [34] and GB/T 50123-2019. Loading was continued until failure and subsequently extended to a deformation of 3 mm, while the load–deformation response was continuously recorded.

The unconfined compressive strength, denoted $q_{u}$ was obtained by dividing the maximum applied load by the initial cross-sectional area of the specimen:

(1) $$q_{u}=\frac{F_{max}}{A_{0}}$$

where $F_{max}$ is the maximum applied load and $A_{0}$ = *a*^2^ is the initial cross-sectional area in square millimeters, with a = 70.7 mm being the specimen side length. Test results deviating by more than 15% from the mean value were excluded prior to averaging.

To quantify the post-peak ductility imparted by fiber reinforcement, the residual load-bearing capacity at 1 mm of deformation beyond the peak was recorded and expressed as a percentage of the peak load. This residual strength ratio, written as $\eta_{r}$, was computed as the post-peak load divided by the peak load, multiplied by 100%:

(2) $$\eta_{r}=\left[\frac{F_{\Delta =1.0}}{F_{max}}\right]\times 100\%$$

where $F_{\Delta =1.0}$ is the load recorded at 1 mm post-peak deformation. Additionally, the ductility index (DI) was computed as the ratio of the total energy absorbed up to 3 mm post-peak deformation to the elastic energy stored up to the peak load, obtained by numerical integration of the stress–strain curve.

Prior to loading, splitting lines were marked on specimen surfaces, and specimens were positioned between steel loading strips measuring 150 mm × 5 mm × 5 mm. Loading was applied at a constant rate of 8 mm/min until failure. The splitting tensile strength, denoted $q_{t}$, was calculated by distributing the failure load over the nominal splitting plane of the cube:

(3) $$q_{t}=\frac{2P}{\pi a^{2}}$$

where *P* is the applied load at failure, and *a* is the specimen side length in millimeters. Outlier treatment followed the same criteria applied to compressive strength results (*n* = 3, 15% exclusion rule). Failure characteristics, including crack development and specimen fragmentation, were documented to compare fiber-reinforced and unreinforced specimens.

#### 2.2.4. Microstructural Analysis

Scanning electron microscopy (SEM) was conducted on selected fractured specimens preserved in anhydrous ethanol to inhibit further hydration. Representative samples with dimensions of approximately 10 mm × 10 mm × 10 mm were prepared by cutting 1 cm^3^ fragments from selected cured samples (70.7 mm cubes), air-drying at 60 °C for 24 h, and sputter-coating with gold to ensure conductivity. The SEM analysis is qualitative in nature, and the interpretations regarding fiber distribution, interfacial bonding, and failure mechanisms should be regarded as observational evidence rather than quantitative proof. Future studies incorporating quantitative image analysis (e.g., fiber dispersion indices) and energy-dispersive X-ray spectroscopy (EDS) are recommended to substantiate these microstructural observations.

#### 2.2.5. Quality Control and Summary

One-way analysis of variance (ANOVA) was conducted separately for each curing age (3, 14, and 28 days) to evaluate the effect of GF content on UCS and STS. Tukey’s honestly significant difference (HSD) test was applied for post hoc pairwise comparisons at a significance level of α = 0.05. A two-way ANOVA (curing age × fiber content) was also performed on the UCS dataset to assess interaction effects across the full experimental design. All individual specimen values, ANOVA tables, and post hoc results are provided in Supplementary Tables S1–S3.

One specimen in the 1.5% GF splitting tensile group (3-day curing) exceeded the ±15% deviation threshold (value: 0.7586 MPa vs. group mean of remaining specimens: 0.6469 MPa, deviation +17.3%) and was excluded from the reported average. No replacement specimen was available; therefore, the reported mean for this specific group is based on *n* = 2. This exclusion is explicitly noted in Supplementary Materials.

## 3. Results

### 3.1. Compressive Strength Development

**Figure 4:**
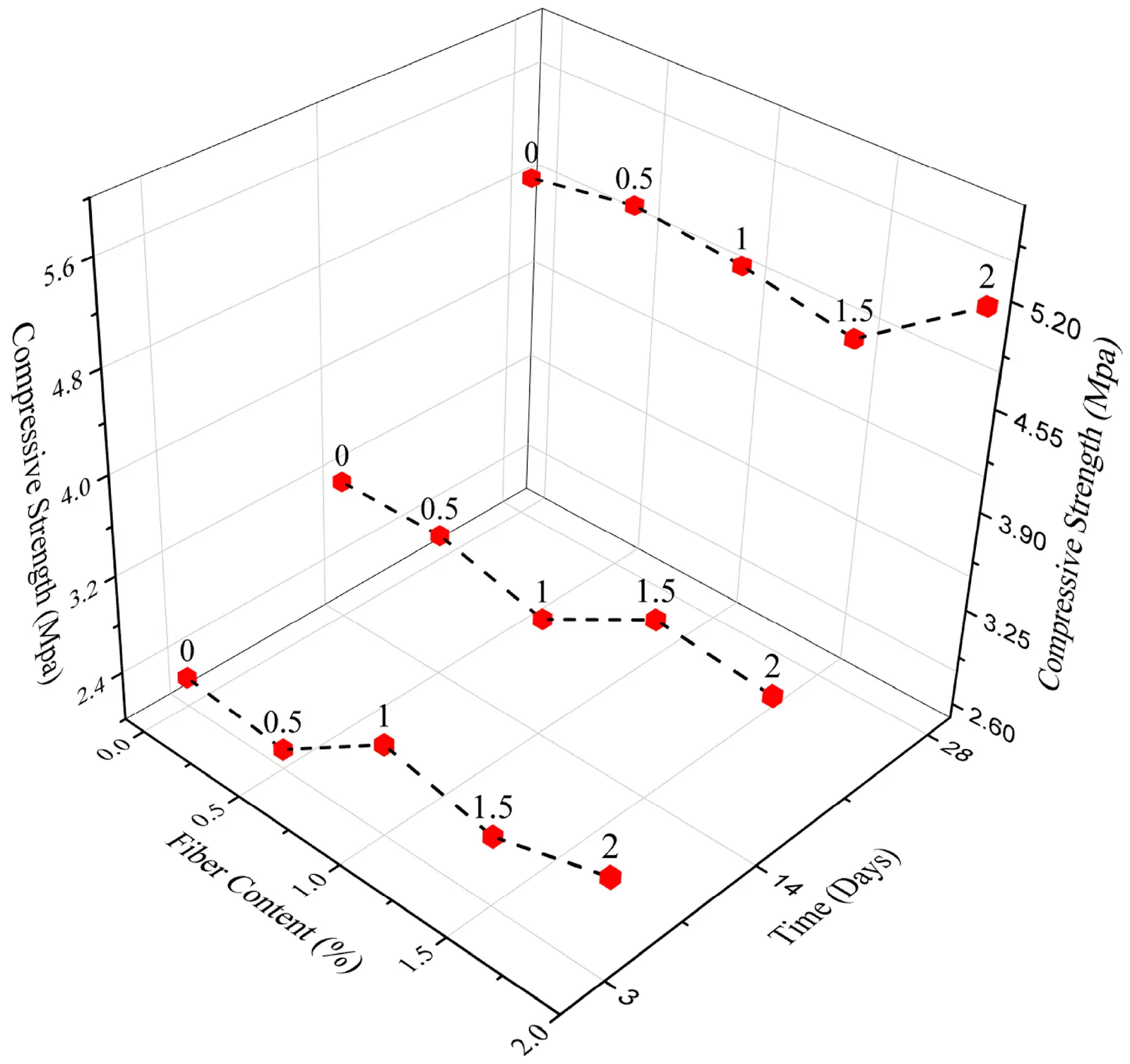
Compressive strength of test blocks at different ages.

The development of unconfined compressive strength (UCS) in the cement-stabilized soil composites, as influenced by glass fiber content and curing age, is presented in Figure 4. All reported values represent the mean of three replicate specimens, with error bars indicating ±1 standard deviation. The coefficient of variation (CV) across all test groups ranged from 4.2% to 11.8%, indicating acceptable repeatability.

Overall, the inclusion of glass fiber enhanced compressive strength at all curing stages, although the extent of improvement depended on fiber content and curing duration. At a fiber content of 0.5%, only limited enhancement was observed across all curing ages. Increasing the fiber content to 1.0% resulted in a pronounced improvement in early-age strength, with the compressive strength of 3-day cured specimens increasing by approximately 15.4% compared with the fiber-free reference mixture. At a fiber content of 1.5%, the most notable improvement occurred at 14 days, where compressive strength increased by approximately 7.3%. The greatest overall enhancement was achieved at a fiber content of 2.0%, corresponding to strength increases of approximately 18.1%, 5.4%, and 9.1% at curing ages of 3, 14, and 28 days, respectively, relative to specimens without glass fiber. Although compressive strength increased with curing time for all mixtures, the relative reinforcing contribution of glass fiber diminished at later ages, indicating that glass fiber addition is more effective in improving the compressive performance of early-age cement-soil when the cementitious matrix is less mature and fiber–matrix interaction plays a more significant role.

**Figure 5:**
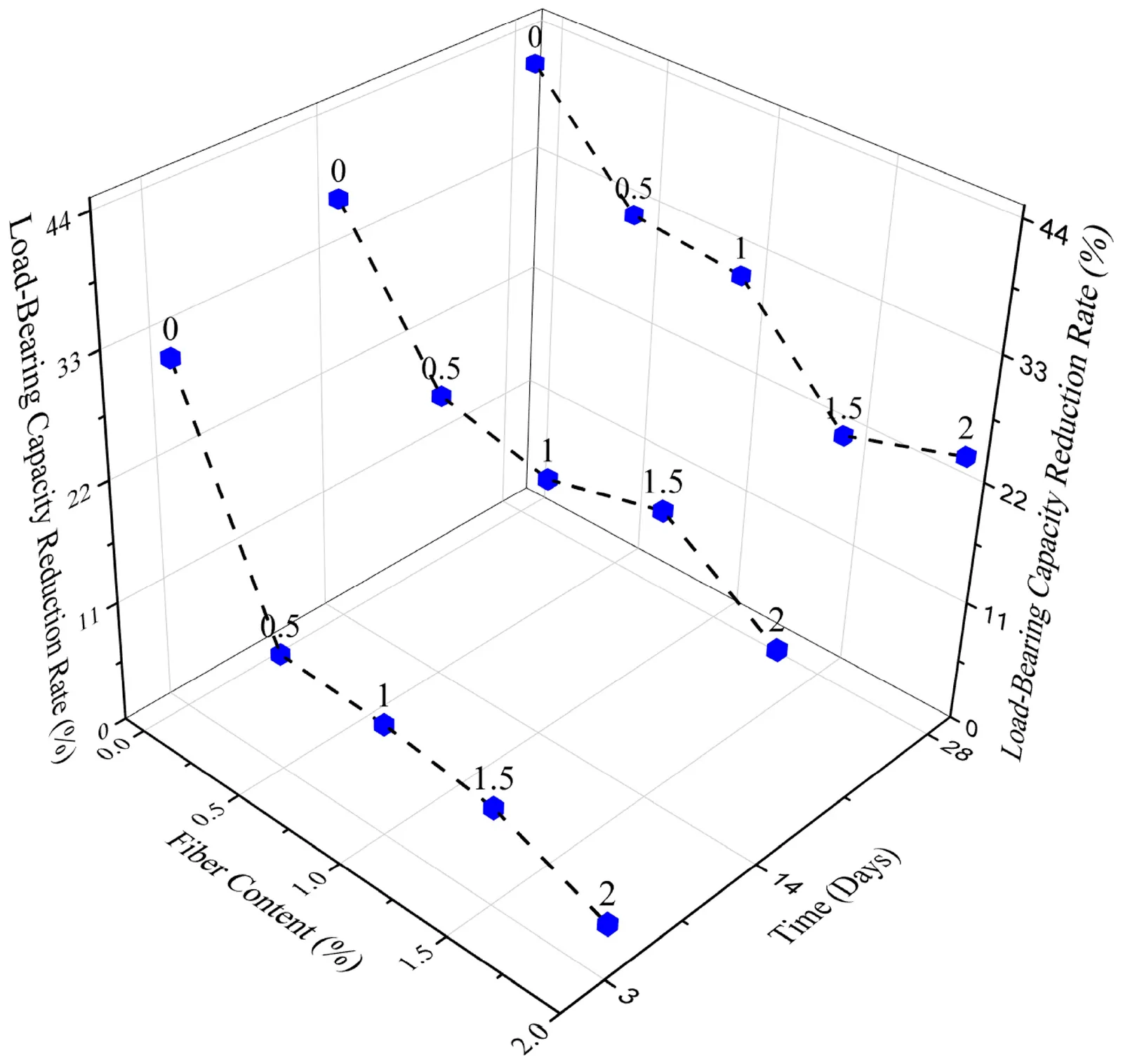
Load capacity degradation rate after specimen failure.

Glass fiber–reinforced specimens exhibited fewer surface cracks with reduced crack widths at failure, whereas unreinforced specimens showed numerous wide cracks accompanied by surface spalling. As shown in Figure 5, the compressive load-bearing capacity of unreinforced specimens declined rapidly with increasing post-peak deformation, reflecting pronounced brittle failure behavior. In contrast, glass fiber–reinforced specimens exhibited a markedly reduced rate of strength degradation after peak load, demonstrating improved post-peak resistance and deformation tolerance.

The residual strength ratio at 1 mm post-peak deformation increased progressively with GF content, from 12.4% for the control to 43.2% at 2.0% GF, representing a 248% improvement in post-peak load-bearing capacity. The ductility index followed a similar trend, increasing from 1.18 to 1.85, confirming the transition from brittle to more ductile failure behavior.

### 3.2. Splitting Tensile Strength Development

**Figure 6:**
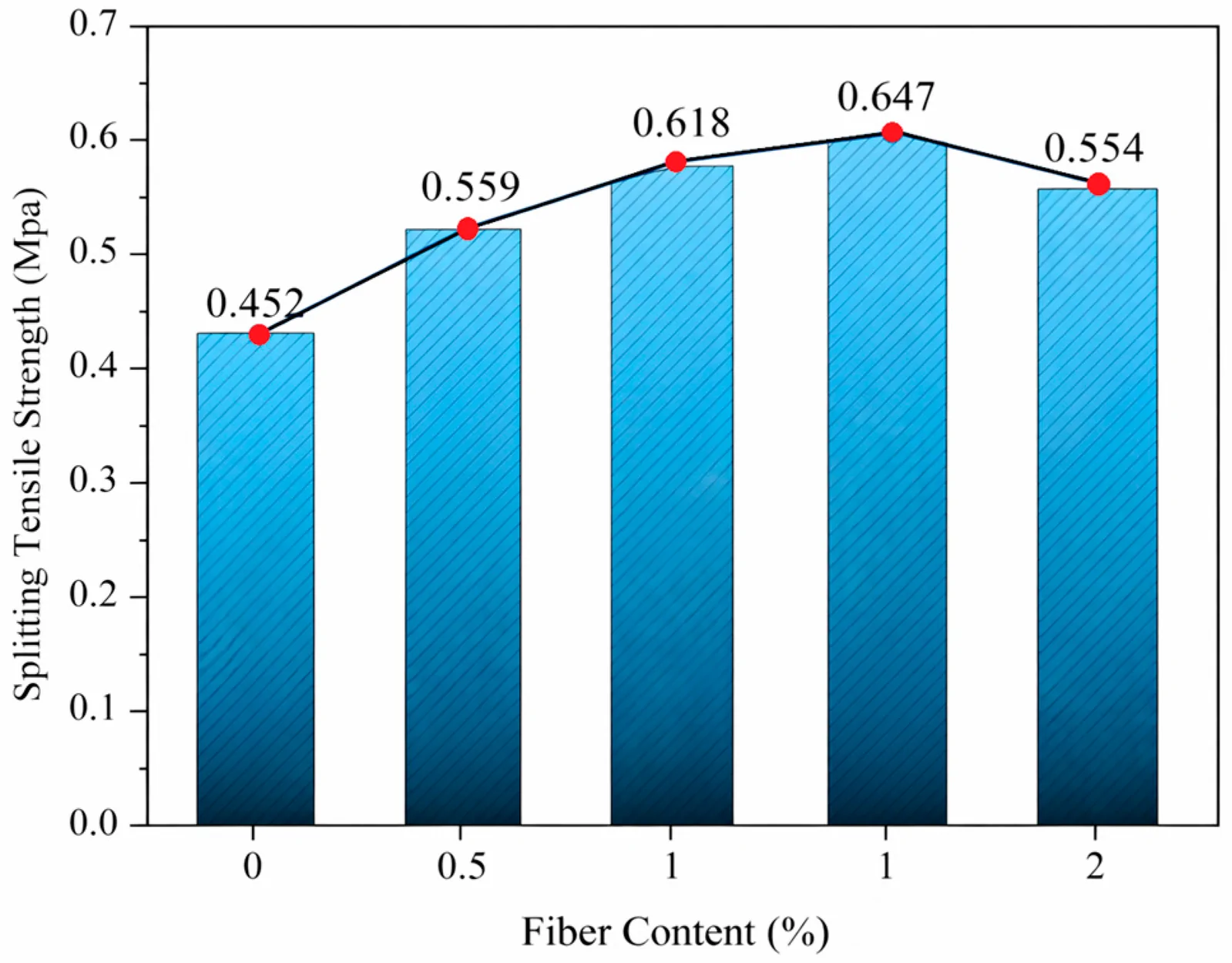
Splitting tensile strength of specimens.

The effectiveness of glass fiber reinforcement in mitigating the low tensile strength of cement-stabilized soil is shown in Figure 6. All values represent mean ± standard deviation (*n* = 3). On average, the splitting tensile strength (STS) increased by approximately 25% across all fiber contents compared with the fiber-free reference mixture.

The improvement was strongly dependent on fiber dosage. At a fiber content of 1.0%, the splitting tensile strength increased by approximately 36%, while a peak enhancement of about 43% was achieved at an optimal fiber content of 1.5%. Further increasing the glass fiber content to 2.0% resulted in a reduced reinforcing effect, with the splitting tensile strength increase declining to approximately 22.6%. This represents a reduction of around 14.3% relative to the peak performance observed at 1.5% fiber content, indicating that excessive fiber addition can adversely affect tensile performance. It should be noted that this trend differs from the compressive strength behavior, for which the highest strength values were generally obtained at a fiber content of 2.0% (Figure 4).

Unreinforced specimens exhibited pronounced brittle failure during splitting tensile testing, typically separating completely into two distinct halves. In contrast, glass fiber–reinforced specimens retained greater structural integrity owing to fiber bridging and bonding effects. Cracks developed without complete separation, and both crack width and depth were significantly reduced compared with unreinforced specimens.

### 3.3. Microstructural Observations

**Figure 7:**
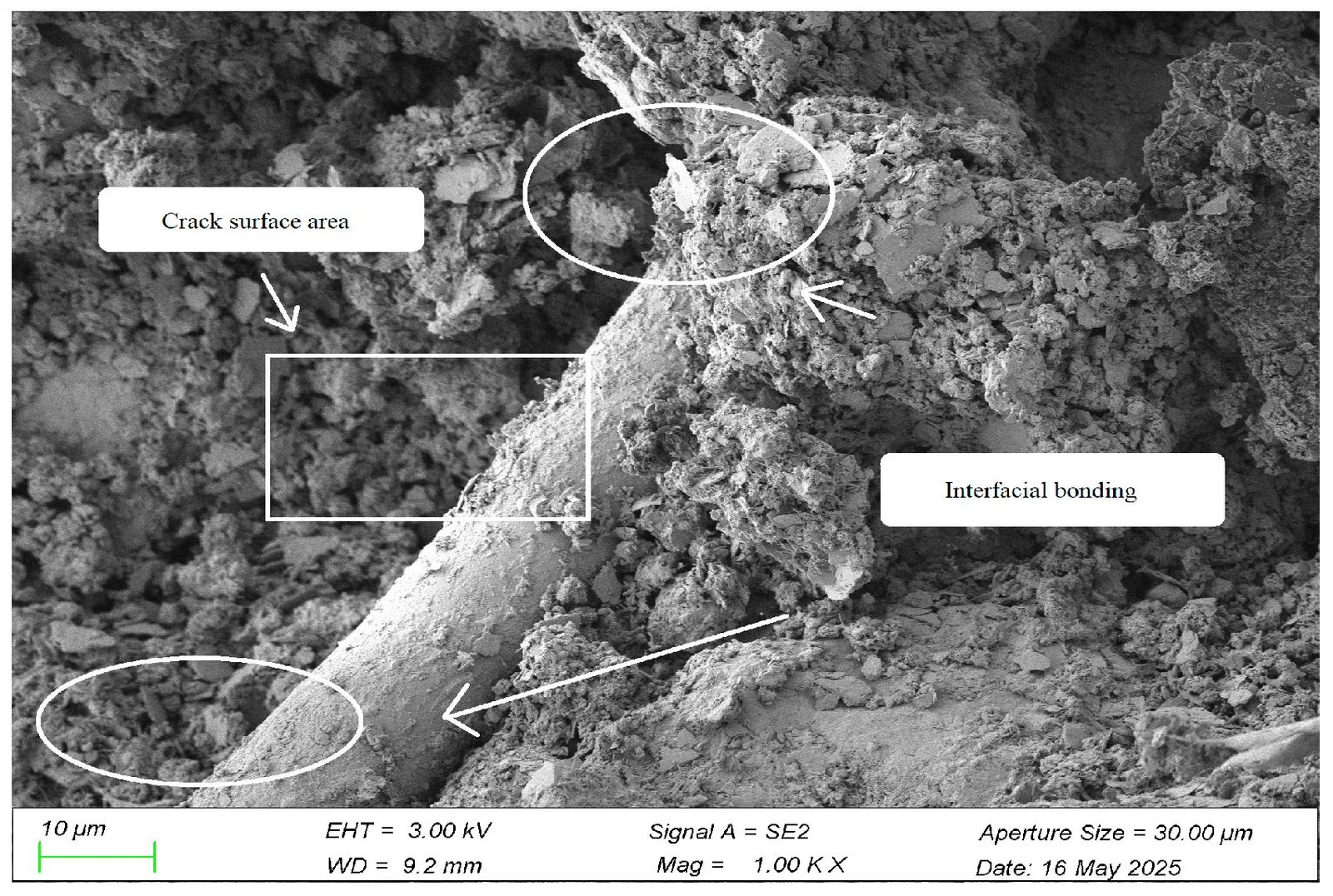
SEM micrograph showing glass fiber bridging a microcrack in cement-stabilized soil (magnification = 1000×).

**Figure 8:**
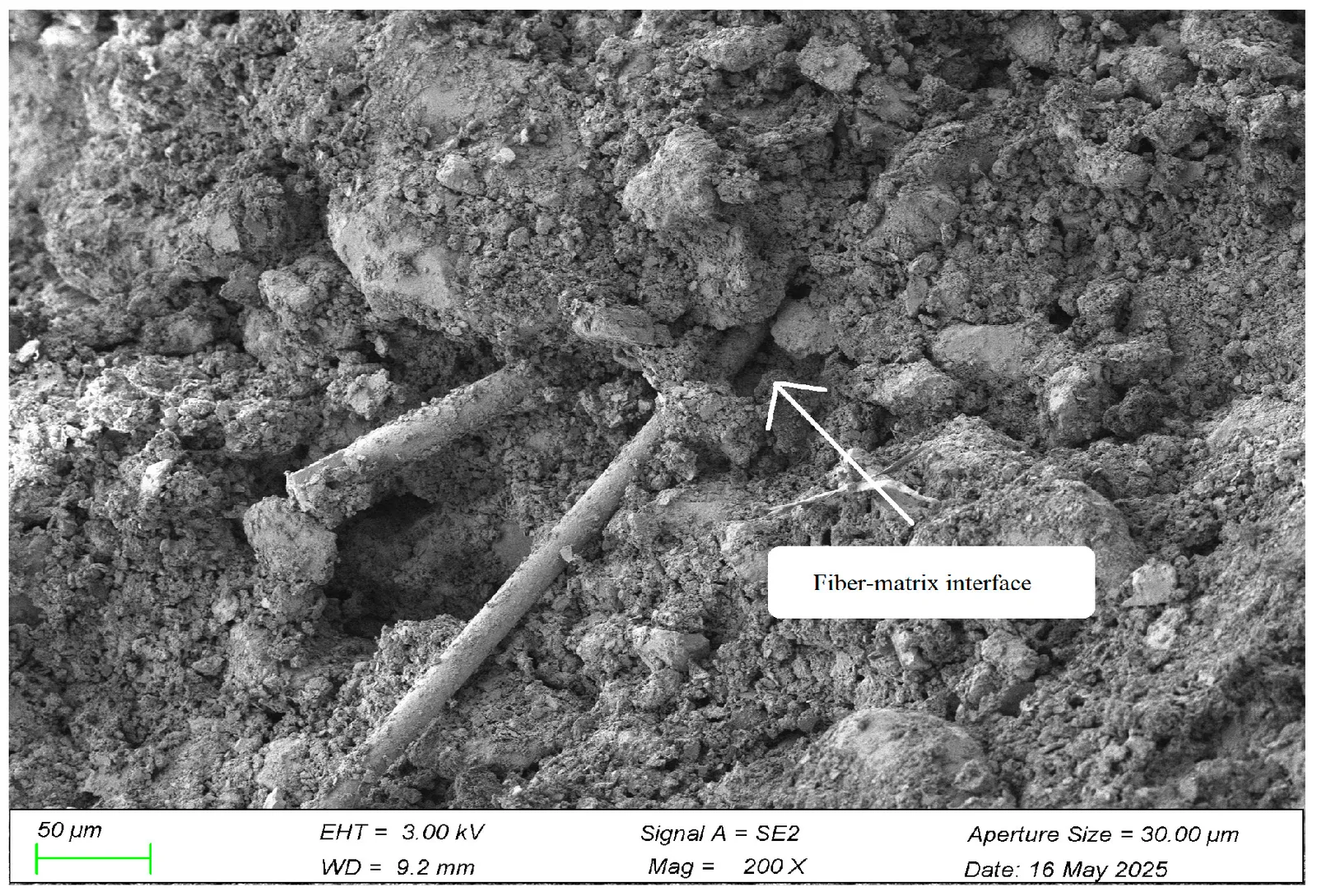
SEM micrograph showing crack propagation along a fiber–matrix interface in GF-modified cement-stabilized soil (magnification = 200×).

Figure 7 shows a glass fiber embedded within the cementitious matrix, bridging a microcrack and anchoring cement-soil particles on both sides of the fracture plane. The fiber restricts crack opening through a combination of frictional and adhesive bond transfer. The glass fibers exhibit both mechanical interlocking and adhesive bonding with hydration products and soil particles, enabling fibers to bridge developing microcracks by connecting particles across fracture surfaces, thereby restricting crack initiation and propagation. As shown in Figure 8, fibers located at crack interfaces during splitting tensile loading are subjected primarily to tensile and shear stresses. Owing to the comparatively high tensile and shear strength of glass fibers relative to the cement-soil matrix, fiber bridging provides an efficient load-transfer mechanism, resulting in a more pronounced improvement in splitting tensile strength than in compressive strength.

Minor variations in compressive strength observed for certain GF-reinforced specimens were attributed to fiber agglomeration arising from excessive fiber content or insufficient mixing. These agglomerates create localized weak zones with reduced interfacial bonding strength, promoting preferential crack initiation under compressive loading. When uniformly dispersed, however, glass fibers form a three-dimensional reinforcement network within the cement-soil matrix that restricts lateral deformation of soil particles and contributes directly to load sharing under compression, thereby enhancing overall strength. Figure 8 illustrates the fiber–matrix adhesion zone after compressive failure, with a crack propagating through the fiber–matrix interface. Localized fiber agglomeration reduces the effective bonding area and creates a preferential failure plane.

**Figure 9:**
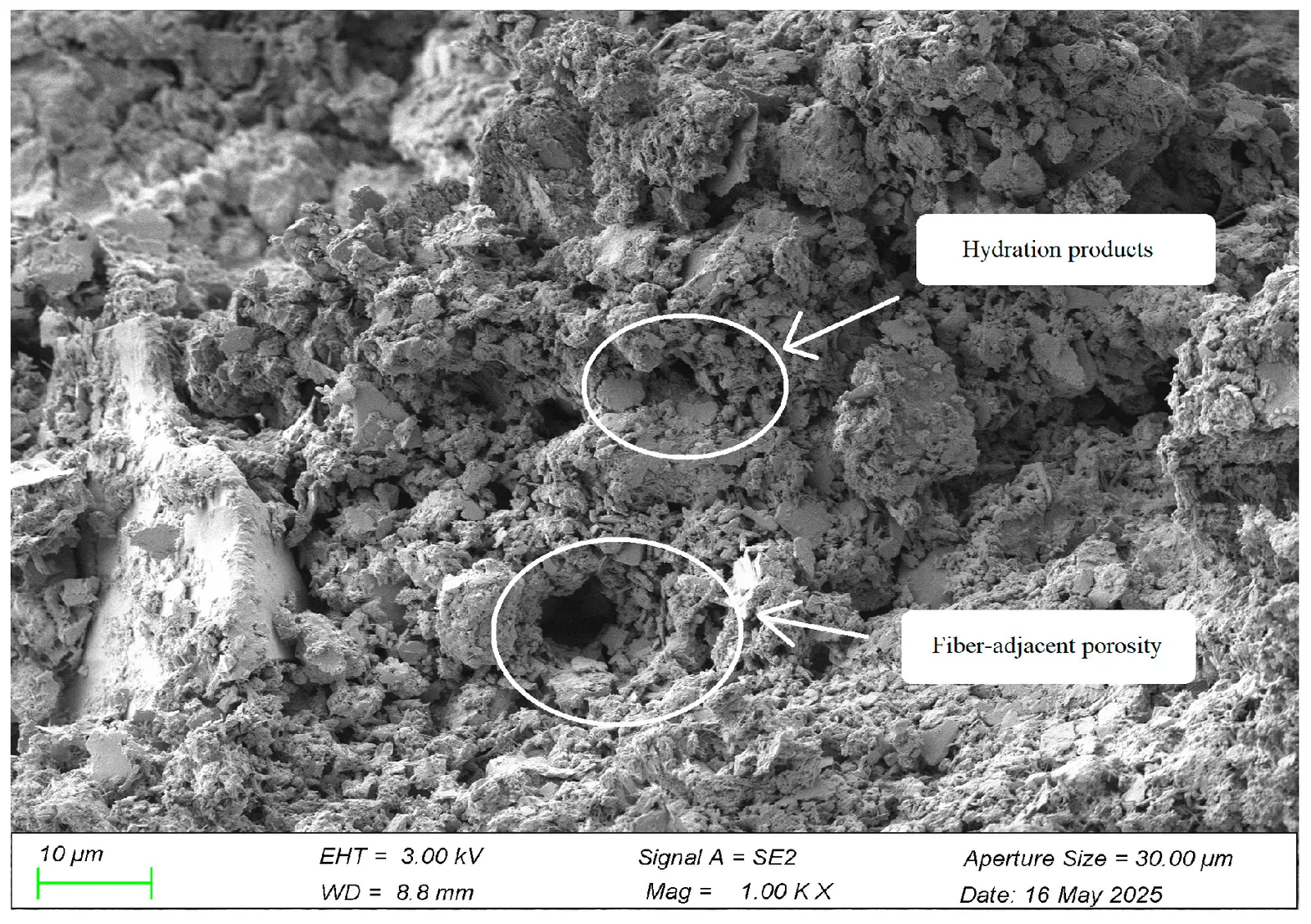
SEM micrograph showing hydration products and fiber-adjacent porosity in GF-modified cement-stabilized soil (magnification = 1000×).

Microstructural observations presented in Figure 9 illustrate the microstructural features of GF-modified cement-stabilized soil. The micrograph shows hydration products and fiber-adjacent porosity surrounding a glass fiber embedded in the matrix. The presence of localized porosity near the fiber–matrix interface may act as a stress concentrator under tensile loading, explaining the reduced splitting tensile strength observed at 2.0% GF where fiber agglomeration and inadequate compaction increase interfacial void content. In contrast, the surrounding dense hydration products confirm that cementitious bonding continues to develop within the matrix. These observations highlight the importance of achieving uniform fiber dispersion and adequate compaction to minimize fiber-adjacent porosity and fully mobilize fiber–matrix adhesion.

## 4. Discussion

Under compressive loading, the three-dimensional GF network restricts lateral particle deformation and redistributes localized stresses throughout the cement-soil matrix, consistent with the fiber-reinforcement mechanisms described by Tamassoki et al. [3] and Xiao et al. [14]. At 2.0% GF, this network remains sufficiently dispersed to contribute to load-bearing capacity under compression, where the confining effect of the surrounding matrix suppresses premature fiber pull-out [4,16]. The post-peak behavior corroborates this mechanism: the residual strength ratio increased from 12.4% to 43.2% and the ductility index from 1.18 to 1.85 at 2.0% GF, indicating that fibers bridge microcracks and maintain structural integrity after peak load [24]. This transformation from brittle fracture to controlled progressive damage confirms that GF incorporation enhances energy dissipation through sequential fiber debonding, elongation, and pull-out [7,8].

In contrast, splitting tensile failure is governed primarily by crack-face bridging and frictional load transfer across propagating cracks [14,28]. At 1.5% GF, fibers are sufficiently dispersed to intercept microcracks efficiently, resulting in a 43% tensile strength increase. However, at 2.0% GF, localized fiber agglomeration reduces the effective interfacial bonding area and creates preferential failure planes along clustered fiber bundles, consistent with the agglomeration-induced weakening reported in fiber-reinforced cementitious systems [4,24]. The SEM micrographs support this interpretation: fiber bridging across cracks was evident in the GF-modified specimens, whereas adhesion zones in higher-fiber specimens exhibited cracks propagating along the fiber–matrix interface. It must be emphasized that these microstructural interpretations are qualitative; quantitative dispersion metrics and energy-dispersive X-ray spectroscopy (EDS) were not employed, and future studies should incorporate quantitative image analysis to substantiate these observations.

From a sustainability perspective, the incorporation of 5% WBP as a partial cement replacement and 1.5–2.0% GF as reinforcement aligns with circular economy principles by valorizing construction waste [9,10,27]. The estimated emission reduction of approximately 85 kg CO_2_eq per cubic meter is based on simplified cradle-to-gate assumptions; however, this preliminary figure excludes WBP grinding energy and GF manufacturing emissions due to data unavailability, and a full ISO 14040/14044 compliant life-cycle assessment is required for definitive quantification [25,29]. Nevertheless, the dual benefit of waste diversion and mechanical enhancement supports the broader ecological transition in construction identified by Liu et al. [29].

Several limitations must be acknowledged. Because WBP was maintained at a fixed 5% replacement ratio while only GF content was varied, the reported optimal dosages apply specifically to the WBP–cement matrix investigated and should not be generalized as combined interaction optima without full factorial experimentation [11]. The sample size (*n* = 3) provides limited statistical power; although the two-way ANOVA confirmed a significant age × fiber interaction (*F*(8, 30) = 2.98, *p* = 0.014), the one-way ANOVAs at 14 days (*p* = 0.071) and 28 days (*p* = 0.137) were marginally non-significant, likely reflecting high within-group variance rather than a true absence of GF effect. All individual specimen values, ANOVA tables, and post hoc results are provided in the Supplementary Materials to ensure full transparency. Furthermore, the study was limited to UCS and STS under standard curing conditions; validation through triaxial shear, cyclic loading, flexural beam, freeze–thaw durability, and long-term alkaline degradation tests is essential before field implementation [19,21]. The dosage recommendations presented herein (2.0% GF for compression-dominated applications, 1.5% GF for tension-critical scenarios) are therefore preliminary and laboratory-based, requiring further experimental and field-scale validation.

## 5. Conclusions

This study investigated the combined modification of cement-stabilized soil with a fixed 5% waste brick powder replacement and variable glass fiber content (0–2.0%). The principal findings are as follows.

- Glass fiber reinforcement enhanced compressive strength most effectively at early ages, with a maximum increase of 18.1% at 2.0% GF and 3 days of curing. The relative reinforcing contribution diminished at later ages of hardening, indicating that GF modification is most advantageous for early-age strength development.
- Splitting tensile strength peaked at 1.5% GF with a 43% increase, whereas 2.0% GF caused a 14.3% reduction relative to this optimum due to fiber agglomeration. This divergence established distinct optimal dosage trends: 2.0% GF under compression-dominated loading and 1.5% GF under tension-critical conditions.
- Fiber incorporation transformed the failure mode from brittle catastrophic fracture to controlled progressive damage. The residual strength ratio increased from 12.4% to 43.2% and the ductility index from 1.18 to 1.85 at 2.0% GF, confirming substantial energy dissipation through fiber debonding, elongation, and pull-out.
- Microstructural observations indicated that uniform fiber dispersion enabled crack bridging and interfacial load transfer, whereas agglomeration created localized weak zones that compromised tensile performance. These interpretations were qualitative and required quantitative image analysis for future validation.
- From a sustainability perspective, the valorization of construction waste through 5% WBP substitution aligned with circular economy principles. Preliminary assessment indicated a potential emission reduction of approximately 85 kg CO_2_eq per cubic meter; however, this estimate excluded WBP processing energy and GF manufacturing emissions, and a full ISO 14040/14044 life-cycle assessment was required for definitive quantification.

It is emphasized that these dosage trends are derived solely from laboratory-scale UCS and STS tests under standard curing conditions. Because WBP was maintained at a fixed replacement ratio, the reported optima apply specifically to the matrix investigated and should not be generalized without full factorial experimentation. Validation through triaxial shear, cyclic loading, flexural beam, freeze–thaw durability, and long-term alkaline degradation tests is essential before field-scale implementation.

## Figures and Tables

**Table 1:** Basic physical properties of soil.

Property	Value
Natural moisture content (%)	13.4
Moisture content of air-dried soil (%)	1.2
Soil density (g cm^−3^)	1.69
Plastic limit (%)	18.0
Liquid limit (%)	27.34

**Table 2:** Physical and mechanical properties of cement.

Property	Value
Specific surface area (m^2^ kg^−1^)	350
Initial setting time (min)	195
Final setting time (min)	250
Soundness	Qualified
3-day flexural strength (MPa)	5.3
3-day compressive strength (MPa)	23.4
Loss on ignition (%)	4.12

**Table 3:** Geometric properties of glass fiber.

Property	Value
Nominal diameter (μm)	7
Length (mm)	6
Aspect ratio (—)	~857

**Table 4:** Mix proportions per batch for three 70.7 mm cubic specimens (1.3 correction factor applied to cementitious materials).

GF Content (%)	Soil (g)	Cement (g)	WBP (g)	GF (g)	Water (g)
0	1851	349	92	0	652
0.5	1851	349	92	9.2	652
1.0	1851	349	92	18.4	652
1.5	1851	349	92	27.6	652
2.0	1851	349	92	36.8	652

## Data Availability

The data presented in this study are available on request from the corresponding author. Some or all data, models, or code generated or used during the study are proprietary or confidential in nature and may only be provided with restrictions.

## Supplementary Materials

The following supporting information can be downloaded at: https://www.mdpi.com/article/10.3390/ma19163410/s1.
