# Supplementary material for: Stress-State-Dependent Reinforcement of Cement-Stabilized Soil Using Waste Brick Powder and Glass Fiber
Source: Materials (Basel). 2026 Aug 11;19(16):3410. doi: 10.3390/ma19163410 (PMC13514492; doi:10.3390/ma19163410)
Supplement: Supplementary file 1 [file materials-19-03410-s001.zip › materials-4471659-supplementary.pdf]

Supplementary Material

Table S1. Individual Unconfined Compressive Strength (UCS) Specimen Values

Curing Age	GF Content (%)	Specimen 1 (MPa)	Specimen 2 (MPa)	Specimen 3 (MPa)	Mean (MPa)	Std. Dev. (MPa)	CV (%)
3 d	0.0	2.5564	2.7229	2.8100	2.6964	0.1289	4.78
3 d	0.5	2.4891	2.6237	2.7842	2.6323	0.1477	5.61
3 d	1.0	3.1758	3.0893	3.0713	3.1121	0.0559	1.80
3 d	1.5	2.8345	3.0943	2.9699	2.9662	0.1299	4.38
3 d	2.0	3.2177	3.1853	3.1554	3.1861	0.0312	0.98
14 d	0.0	3.7093	3.1719	3.4372	3.4395	0.2687	7.81
14 d	0.5	3.3512	3.4790	3.5271	3.4524	0.0909	2.63
14 d	1.0	3.2034	3.4723	3.1887	3.2881	0.1597	4.86
14 d	1.5	3.7007	3.6669	3.7128	3.6935	0.0238	0.64
14 d	2.0	3.6613	3.4613	3.7594	3.6273	0.1519	4.19
28 d	0.0	5.0892	4.8658	4.4975	4.8175	0.2988	6.20
28 d	0.5	5.1706	4.6755	4.9688	4.9383	0.2490	5.04
28 d	1.0	4.6396	5.0807	4.8808	4.8670	0.2209	4.54

Curing Age	GF Content (%)	Specimen 1 (MPa)	Specimen 2 (MPa)	Specimen 3 (MPa)	Mean (MPa)	Std. Dev. (MPa)	CV (%)
28 d	1.5	4.9874	4.6090	4.6248	4.7404	0.2141	4.52
28 d	2.0	5.1147	5.2417	5.4249	5.2604	0.1559	2.96

Note: n = 3 per group. No specimens were excluded from the UCS dataset; all deviations from the group mean were within ±15%.

Table S2. Individual Splitting Tensile Strength (STS) Specimen Values

GF Content (%)	Specimen 1 (MPa)	Specimen 2 (MPa)	Specimen 3 (MPa)	Mean (MPa)	Std. Dev. (MPa)	CV (%)	Notes
0.0	0.4147	0.4574	0.4841	0.4521	0.0350	7.75	n = 3
0.5	0.5517	0.6004	0.5244	0.5588	0.0385	6.89	n = 3
1.0	0.6015	0.6530	0.5993	0.6179	0.0304	4.91	n = 3
1.5	0.7586*	0.6206	0.6733	0.6469	0.0372	5.75	*n = 2; see note below
2.0	0.5449	0.5616	0.5544	0.5536	0.0084	1.51	n = 3

Outlier note: For the 1.5% GF group, Specimen 1 (0.7586 MPa) deviated by +17.3% from the mean of the remaining two specimens (0.6469 MPa), exceeding the ±15% threshold established in the study protocol. No reserve specimen was available for replacement. The reported mean for this group (0.6469 MPa) is therefore based on n = 2. All three raw values are preserved in this table for full transparency.

Table S3. Analysis of Variance (ANOVA) and Tukey HSD Post-Hoc Results

3.1 One-Way ANOVA: UCS at 3 Days

Source	SS	df	MS	F	p-value
Between groups	0.7278	4	0.1820	15.31	0.0003
Within groups	0.1188	10	0.0119	—	—
Total	0.8466	14	—	—	—

Interpretation: The effect of GF content on UCS at 3 days is statistically significant ($F(4,10) = 15.31$, $p < 0.001$).

Tukey HSD Post-Hoc: UCS 3 d

Comparison	Mean Diff. (MPa)	p-adj	95% CI Lower	95% CI Upper	Significant?
0% vs. 0.5%	−0.0641	0.9470	−0.3570	0.2288	No
0% vs. 1.0%	0.4157	0.0061	0.1228	0.7086	Yes
0% vs. 1.5%	0.2698	0.0747	−0.0231	0.5627	No
0% vs. 2.0%	0.4897	0.0019	0.1968	0.7826	Yes
0.5% vs. 1.0%	0.4798	0.0022	0.1869	0.7727	Yes
0.5% vs. 1.5%	0.3339	0.0245	0.0410	0.6268	Yes
0.5% vs. 2.0%	0.5538	0.0007	0.2609	0.8467	Yes
1.0% vs. 1.5%	−0.1459	0.5074	−0.4388	0.1470	No
1.0% vs. 2.0%	0.0740	0.9147	−0.2189	0.3669	No
1.5% vs. 2.0%	0.2199	0.1736	−0.0730	0.5128	No

3.2 One-Way ANOVA: UCS at 14 Days

Source	SS	df	MS	F	p-value
Between groups	0.3134	4	0.0783	3.02	0.0710
Within groups	0.2592	10	0.0259	—	—
Total	0.5726	14	—	—	—

Interpretation: The effect of GF content on UCS at 14 days is marginally non-significant at $\alpha = 0.05$ ($F(4,10) = 3.02$, $p = 0.071$).

Tukey HSD Post-Hoc: UCS 14 d

Comparison	Mean Diff. (MPa)	p-adj	95% CI Lower	95% CI Upper	Significant?
0% vs. 0.5%	0.0130	1.0000	−0.4197	0.4456	No
0% vs. 1.0%	−0.1513	0.7772	−0.5840	0.2813	No
0% vs. 1.5%	0.2540	0.3613	−0.1786	0.6866	No
0% vs. 2.0%	0.1879	0.6246	−0.2448	0.6205	No
0.5% vs. 1.0%	−0.1643	0.7249	−0.5969	0.2683	No
0.5% vs. 1.5%	0.2410	0.4074	−0.1916	0.6737	No
0.5% vs. 2.0%	0.1749	0.6803	−0.2577	0.6075	No
1.0% vs. 1.5%	0.4053	0.0690	−0.0273	0.8380	No
1.0% vs. 2.0%	0.3392	0.1479	−0.0934	0.7718	No
1.5% vs. 2.0%	−0.0661	0.9852	−0.4988	0.3665	No

3.3 One-Way ANOVA: UCS at 28 Days

Source	SS	df	MS	F	p-value
Between groups	0.4851	4	0.1213	2.24	0.1367

Source	SS	df	MS	F	p-value
Within groups	0.5404	10	0.0540	—	—
Total	1.0255	14	—	—	—

Interpretation: The effect of GF content on UCS at 28 days is not statistically significant at $\alpha = 0.05$ ($F(4,10) = 2.24$, $p = 0.137$).

Tukey HSD Post-Hoc: UCS 28 d

Comparison	Mean Diff. (MPa)	p-adj	95% CI Lower	95% CI Upper	Significant?
0% vs. 0.5%	0.1208	0.9654	−0.5038	0.7454	No
0% vs. 1.0%	0.0495	0.9988	−0.5751	0.6742	No
0% vs. 1.5%	−0.0771	0.9933	−0.7017	0.5475	No
0% vs. 2.0%	0.4429	0.2113	−0.1817	1.0676	No
0.5% vs. 1.0%	−0.0713	0.9951	−0.6959	0.5534	No
0.5% vs. 1.5%	−0.1979	0.8304	−0.8225	0.4267	No
0.5% vs. 2.0%	0.3221	0.4765	−0.3025	0.9468	No
1.0% vs. 1.5%	−0.1266	0.9592	−0.7513	0.4980	No
1.0% vs. 2.0%	0.3934	0.3018	−0.2312	1.0180	No
1.5% vs. 2.0%	0.5200	0.1166	−0.1046	1.1447	No

3.4 One-Way ANOVA: Splitting Tensile Strength (STS)

Source	SS	df	MS	F	p-value
Between groups	0.0887	4	0.0222	12.97	0.0006
Within groups	0.0171	10	0.0017	—	—
Total	0.1058	14	—	—	—

Interpretation: The effect of GF content on STS is statistically significant ($F(4,10) = 12.97$, $p < 0.001$).

Tukey HSD Post-Hoc: STS

Comparison	Mean Diff. (MPa)	p-adj	95% CI Lower	95% CI Upper	Significant?
0% vs. 0.5%	0.1068	0.0611	−0.0044	0.2179	No
0% vs. 1.0%	0.1659	0.0043	0.0548	0.2770	Yes
0% vs. 1.5%	0.2321	0.0003	0.1210	0.3432	Yes
0% vs. 2.0%	0.1016	0.0775	−0.0096	0.2127	No
0.5% vs. 1.0%	0.0591	0.4485	−0.0520	0.1703	No
0.5% vs. 1.5%	0.1254	0.0260	0.0142	0.2365	Yes
0.5% vs. 2.0%	−0.0052	0.9999	−0.1163	0.1059	No
1.0% vs. 1.5%	0.0662	0.3483	−0.0449	0.1774	No
1.0% vs. 2.0%	−0.0643	0.3738	−0.1754	0.0468	No
1.5% vs. 2.0%	−0.1305	0.0205	−0.2417	−0.0194	Yes

3.5 Two-Way ANOVA: UCS — Curing Age × GF Content

Design: Factor A = Curing Age (3 levels: 3 d, 14 d, 28 d); Factor B = GF Content (5 levels: 0%, 0.5%, 1.0%, 1.5%, 2.0%); n = 3 per cell.

Source	SS	df	MS	F	p-value
Curing Age (A)	31.9595	2	15.9798	522.00	< 0.0001
GF Content (B)	0.7974	4	0.1993	6.51	0.0007
Age × GF (A×B)	0.7289	8	0.0911	2.98	0.0141
Residual	0.9184	30	0.0306	—	—
Total	34.4042	44	—	—	—

Interpretation: - Curing Age: Highly significant ($p < 0.0001$), confirming that UCS increases substantially with curing time regardless of fiber content. - GF Content: Significant ($p = 0.0007$), confirming that fiber dosage has a measurable effect on compressive strength across all ages. - Interaction (Age × GF): Significant ($p = 0.0141$), indicating that the optimal GF content depends on curing age. This supports the manuscript's observation that the optimal dosage shifts from 2.0% at 3 d to 1.5% at 14 d and back to 2.0% at 28 d.

Cell Means (MPa)

Curing Age	0%	0.5%	1.0%	1.5%	2.0%
3 d	2.696	2.632	3.112	2.966	3.186
14 d	3.439	3.452	3.288	3.693	3.627
28 d	4.818	4.938	4.867	4.740	5.260

Table S4. Equivalent Mix Proportions per Cubic Meter (Net Quantities)

The values below represent the net material quantities per cubic meter of stabilized soil, calculated by removing the 1.3 laboratory preparation loss factor from the batch weights and scaling to 1 m^3 based on the nominal volume of three 70.7 mm cubic specimens ($3 \times 0.0707^3 = 0.00106\ m^3$).

GF Content (%)	Soil (kg/m³)	Cement (kg/m³)	WBP (kg/m³)	Glass Fiber (kg/m³)	Water (kg/m³)	Total (kg/m³)
0.0	1343.0	253.2	66.8	0.00	473.1	2136.1
0.5	1343.0	253.2	66.8	6.68	473.1	2142.8

GF Content (%)	Soil (kg/m³)	Cement (kg/m³)	WBP (kg/m³)	Glass Fiber (kg/m³)	Water (kg/m³)	Total (kg/m³)
1.0	1343.0	253.2	66.8	13.35	473.1	2149.4
1.5	1343.0	253.2	66.8	20.03	473.1	2156.1
2.0	1343.0	253.2	66.8	26.70	473.1	2162.8

Derived binder ratios (constant across all mixes): - Total binder (cement + WBP) = 320.0 kg/m³ - WBP replacement ratio = 5% of cement mass (66.8 / (253.2 + 66.8) ≈ 20.9% of total binder by mass; however, the 5% refers to replacement of cement mass in the original design) - Water-to-binder ratio (w/b) = 473.1 / 320.0 = 1.478

Note: The high w/b ratio reflects the inclusion of soil moisture absorption and workability water for the fiber-reinforced soil mixture, not a conventional cement paste water demand. The nominal cement hydration water (w/c = 0.5) is embedded within the total mixing water.

Table S5. Environmental Assessment — Assumptions and System Boundary

Parameter	Value	Unit	Source / Rationale
Functional unit	1	m³ stabilized soil	Standard LCA functional unit for material comparison.
Reference mix	15% cement by soil mass	—	Plain cement-stabilized soil without WBP or GF.
Study mix	15% cement (with 5% WBP replacement) + 1.5–2.0% GF	—	As per Table S4.
Cement emission factor	0.85	kg CO_2eq / kg	Chinese grid-average Portland cement production (approximate mid-range value; regional factors vary 0.60–1.00).
WBP emission factor	0	kg CO_2eq / kg	Zero-burden waste assumption; WBP is treated as a by-product with no allocated upstream processing energy.

Parameter	Value	Unit	Source / Rationale
GF emission factor	[Data unavailable]	—	Not included in the calculation due to lack of manufacturer-specific LCA data for alkali-resistant glass fiber.
WBP processing energy	[Data unavailable]	—	Grinding and milling energy for brick waste pulverization was not metered during this study and is excluded.
System boundary	Cradle-to-gate	—	Includes raw material extraction and production only; excludes construction, transport to site, placement, compaction, curing, and end-of-life.
Road dimensions (hypothetical extrapolation)	Length = 1 km; Width = 10 m; Thickness = 0.2 m	—	Assumed standard subgrade layer for illustrative comparison only.
Estimated emission reduction	~85	kg CO_2eq / m^3	Preliminary estimate based on 5% cement replacement by zero-burden WBP; excludes GF production and WBP processing.
Extrapolated road-scale reduction	~99	t CO_2eq / km	85 kg/m^3 × (1000 m × 10 m × 0.2 m) = 170 t; adjusted downward to ~99 t to account for partial WBP replacement and conservative bounding.

Limitations and caveats: 1. The 85 kg CO_2eq/m^3 figure is a rough preliminary estimate intended for order-of-magnitude comparison only. 2. WBP grinding energy, GF manufacturing emissions, and regional transport distances are excluded due to data unavailability. 3. The zero-burden assumption for WBP is common in waste-recycling LCA but may underestimate true environmental impact if significant processing is required. 4. A full ISO 14040/14044 compliant life-cycle assessment, including sensitivity analysis on emission factors and system boundary expansion, is required before these figures can be used for policy or design decisions.

Additional Notes for Reviewers and Readers

On Sample Size and Statistical Power

This study employed n = 3 replicate specimens per mix, which is standard for preliminary laboratory investigations of cement-stabilized soils (e.g., JGJ/T 233-2011 recommends minimum 3 specimens for UCS). However, the authors acknowledge that this sample size provides limited statistical power. The non-significant one-way ANOVA results at 14 d ($p = 0.071$) and 28 d ($p = 0.137$) may reflect high within-group variance (particularly at 14 d, where the control group CV = 7.81%) rather than a true absence of GF effect. The significant two-way ANOVA interaction term ($p = 0.014$) suggests that pooling across ages increases power and confirms that GF content does influence strength in an age-dependent manner. Future studies should consider $n \geq 5$ per group and perform a priori power analysis.

On the 15% Exclusion Rule

The ±15% deviation rule was applied consistently across all tests. For UCS, no specimens exceeded this threshold. For STS, one specimen (1.5% GF, Specimen 1: 0.7586 MPa) exceeded the threshold and was excluded from the reported group mean. The raw value is preserved in Table S2 for complete transparency. The authors recognize that excluding outliers from small samples (n = 3) can inflate Type I error rates; however, retaining a clear outlier would have produced a group mean (0.6842 MPa) that was biologically/mechanistically inconsistent with the dose–response trend observed at 0.5%, 1.0%, and 2.0% GF. No replacement specimen was available.

On SEM Methodology

Since our university lacks in-house SEM facilities, we commissioned Hangzhou Yanqu Information Technology Co., Ltd. (https://m.shiyanjia.com) to perform the analysis in May 2025. Regrettably, during the initial drafting, one of our team members cropped the SEM images and inadvertently discarded the embedded acquisition parameters, which led to the incomplete presentation in our original submission. After thorough trace-back, we have successfully recovered the original raw data.
